# Metabolomic Profiles for HBV Related Hepatocellular Carcinoma Including Alpha-Fetoproteins Positive and Negative Subtypes

**DOI:** 10.3389/fonc.2019.01069

**Published:** 2019-10-15

**Authors:** Jianping Sun, Yanan Zhao, Ling Qin, Kang Li, Yan Zhao, Huanqin Sun, Ting Zhang, Yonghong Zhang

**Affiliations:** ^1^Beijing You'an Hospital, Capital Medical University, Beijing, China; ^2^NHC Key Laboratory of Systems Biology of Pathogens, Institute of Pathogen Biology, Chinese Academy of Medical Sciences and Peking Union Medical College, Beijing, China

**Keywords:** hepatocellular carcinomas, metabolomics, alpha-fetoproteins, biomarkers, HBV

## Abstract

**Background:** Hepatocellular carcinoma (HCC) is very common globally prevalent cancer. Due to its poor clinical prognosis, increasing the diagnostic rate of HCC is urgently needed. Herein, we validate discovered metabolomic biomarkers to distinguish Hepatitis B virus (HBV)-related HCC, including alpha-fetoprotein (AFP) negative (AFP–) and positive (AFP+) individuals.

**Methods:** We recruited 130 HCC subjects (independent case-control, randomized clinical cohorts) to our study. We separated the subjects randomly into two panels: (1) 58 individuals for the discovery panel; and (2) 72 individuals for the validation panel. For each panel, gender and age-matched hepatitis B group (HBG) and healthy group were included as controls. Plasma samples were collected for metabolic profiling by liquid chromatography—mass spectrometry—based metabolomics assays. We applied both non-targeted metabolomics analyses and targeted metabolomics analyses. Significantly changed metabolites (SCMs) were identified. The power of SCMs to discriminate HCC and HBG or healthy group was determined by receiver operating characteristic curve (ROC) analysis.

**Results:** Ten SCMs were selected form the discovery panel, and further verified in the validation panel. ROC analyses indicated that 1 SCMs (LysoPC (24:0)) could discriminate HCC from HBG (AUC = 0.765). Further, 8 SCMs including (LysoPC (17:0), LysoPC (20:4(8Z,11Z,14Z,17Z)), LysoPC (22:0), LysoPC (24:0), PE (P-16:0/22:4(7Z,10Z,13Z,16Z)), SM (d18:1/22:1(13Z)), Creatinine, and L-Isoleucine) displayed a heightened ability to discriminate between HCC and healthy controls (AUC were more than 0.800). Most of these SCMs were important in lipid metabolism.

**Conclusions:** LysoPC (24:0) could distinguished HCC from HBG, and 8 SCMs distinguished HCC from healthy controls. LysoPC and other metabolites have the potential to serve as non-invasive biomarkers for HBV related AFP– and AFP+ HCC.

## Introduction

Hepatocellular carcinoma (HCC) is the third leading cause of cancer mortality in the world. About 278.07/100000 new cases, and 167.89/100000 deaths were reported each year in China (1). Hepatitis B virus (HBV) is the most common cause of HCC, accounting for more than 50% of total mortality (2–4). HCC diagnosed at a late, advanced stage of progression, and usually with ineffective therapies being available, which contributes to the high mortality rate in HCC (5). In addition, a lack of reliable biomarkers to diagnose HCC remains a significant challenge.

Alpha-Fetoprotein (AFP), which was first identified in the 1970s, has often been used as a blood biomarker for HCC (6). However, the sensitivity of AFP is limited to 65% for clinical HCC diagnoses and <40% for preclinical predictions (7–9). The sensitivity and specificity of AFP are not fully applicable to clinical use at this time. For AFP negative (AFP–) HCC, liver biopsy is the current gold standard in clinical diagnosis; however, as an invasive diagnostic approach, it suffers from many limitations, and a non-invasive diagnostic tool is required. Therefore, it is necessary to develop reliable biomarkers to further strengthen the goal of increasing the diagnosis rate in HCC.

Metabolomics represents a rapidly emerging approach to study small molecules (i.e., molecular weight <1,800 Da) that define the metabolic status of a biological system. Such small molecules play important roles in biological systems. Thus, metabolomic profiling has been used to explore the discovery of useful early biomarkers in many human diseases (10). Metabolomics has the potential to precisely measure the metabolite complement in living systems, and can capture the global dynamic responses to changes that from both endogenous and exogenous factors. Metabolomics clearly provides us with many potential applications and advantages for research into complex systems, and a useful platform to detect and study dysregulated metabolic pathways in the field of HCC genesis (11).

It has been reported that a serological panel of metabolite biomarkers exhibits good diagnostic performance in the early detection of HCC as compared detecting the levels of AFP (12). Through a metabolomics approach, Wang et al. described the identification of 13 potential biomarkers and corresponding pathways that were significantly apparent in patients with HCC (10). Of the candidate biomarkers, glycochenodeoxycholic acid was proposed as a potential indicator for HCC diagnosis (13). Although, there are many studies striving to identify new biomarkers for HCC by metabolomics approaches (10–13), the majority of these studies have not included AFP–HCC as a study group. In this context, we have recruited gender- and age-matched AFP negative (AFP–) HCC and compared this group with AFP positive (AFP+) HCC, with the over-arching aim of identifying metabolomic biomarkers that are characteristic of HCC.

Moreover, to exclude the effects of HCC therapy, we chose HCC that never received HCC therapy when recruited. Our study quantified metabolomic profiles in plasma samples amongst the different groups, and to discover diagnostic biomarkers for HCC, a special metabolic pathway is also included.

## Materials and Methods

### Clinical Design and Sample Collection

One hundred and thirty individuals participated in this study at Beijing You'an Hospital of the Capital Medical University from 2014 to 2017, in a case-control, randomized clinical cohort study. Diagnosis of HCC and chronic hepatitis B were based on the EASL Clinical Practice Guidelines (14) and the AASLD practice guidelines (15). Entry criteria included: (1) being diagnosed as HCC by liver biopsy or CT/MRI; (2) individuals with positive HBV surface antigen (HBsAg). Exclusion criteria included: (1) individuals undergoing treatment; for example, interventional surgery, radiofrequency ablation, liver transplantation, TACE, liver cancer resection, and any other treatments for HCC; (2) other hepatitis virus infections; (3) combined presentation of metabolic related disease, including diabetes or cardiovascular diseases; (4) combined presentation of autoimmune-related disease; (5) individuals with a recurrent tumor.

Next, the level of AFP (Roche, Cobas e601, Germany) in the AFP–HCC group was normal (AFP ≤ 7 ng/ml), and the level of AFP in the AFP+HCC group was AFP > 20 ng/ml. Based on the criteria, HCC individuals were separated into a discovery panel and a validation panel according to their age (40–50 years for the discovery panel and 50–70 years for the validation panel). The gender- and age-matched hepatitis B group (HBG) and healthy groups were recruited as controls for this study and were randomly separated into the discovery and validation panels.

All individuals were subjected to the written informed consent requirement, and the local ethics committee of Beijing You'an hospital, Capital Medical University approved this study. The plasma samples were collected immediately after being recruited, following which, the levels of HBsAg, hepatitis B virus surface antibody, hepatitis B virus e antigen, hepatitis B virus e antibody, hepatitis B virus core antibody, HBV DNA, liver function and blood routine analyses were measured. All plasma samples were stored at −80°C until use.

### Plasma Sample Preparation

Plasma samples were thawed on ice at 4°C for 30–60 min, then vortexed for a few seconds and centrifuged at 12,000 rpm for 10 min at 4°C. Aliquots of 100 μl plasma were placed into labeled microcentrifuge tubes for analysis. Pooled quality control samples (QCs) consisted of small aliquots of each biological sample being pooled, which were mixed together to monitor the stability and reproducibility of the results.

### Screening Metabolite Profiles of Different Groups

**Figure 1:**
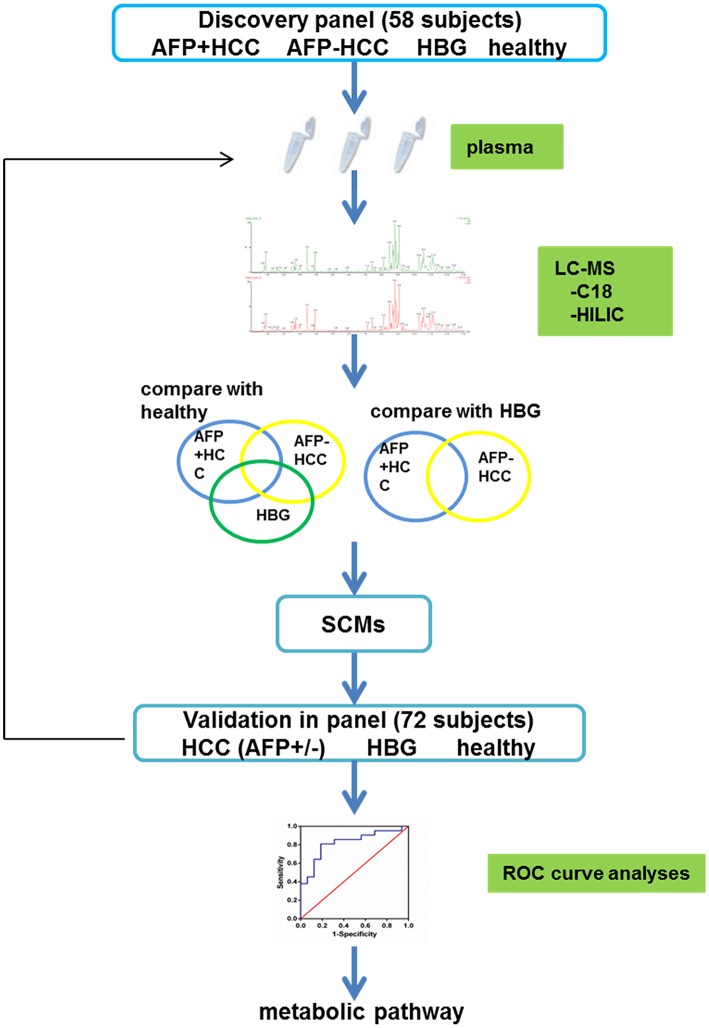
The diagram illustrating the study design.

The metabolites in the discovery panel including 58 individuals (Figure 1), were screened using the Thermo Scientific™ Dionex™ UltiMate™ 3000 Rapid Separation LC (RSLC) system. A reversed phase C18 column and hydrophilic interaction liquid chromatography column (HILIC) were used for UHPLC separations (positive and negative mode ions combined).

### RP Separation for Lipids

For the C18 separation, mobile phase A was acetonitrile/water (60/40) and mobile phase B was isopropanol/acetonitrile (90/10). Both mobile phase A and B contained 0.1% formic acid and 10 mmol/L ammonium acetate. Gradient conditions for reversed phase C18 separation is shown in Table S1. The column was HSS T3 (2.1 × 100 mm, 1.8 μm, water) that was operated at 45°C. The flow rate was 300 μL/min and the injection volume was 1 μL.

### HILIC Separation

For the HILIC separation, mobile phase A was acetonitrile and mobile phase B was water; both mobile phase A and B contained 0.1% formic acid and 10 mmol/L ammonium acetate. The column was a BEH amide column (2.1 × 100 mm, 1.7 μm, water) that was operated at 40°C. The flow rate was 300 μL/min and the injection volume was 1 μL. The gradient conditions for HILIC separation is shown in Table S1.

### Data Acquisition

After the chromatographic separations, using a Thermo Scientific™ Q Exactive™ hybrid quadrupole Orbitrap mass spectrometer equipped with a HESI-II probe, we could perform data acquisition. The positive and negative HESI-II spray voltages were 3.7 and 3.5 kV, respectively. The heated capillary temperature was set to 320°C. The sheath gas pressure was 30 psi. The auxiliary gas setting was 10 psi, and the heated vaporizer temperature was 300°C. Both the sheath gas and the auxiliary gas were nitrogen. The collision gas was also nitrogen at a pressure of 1.5 mTorr. The parameters of the full mass scan were as follows: a resolution of 70,000, an auto gain control target that was under 1 × 10^6^, a maximum isolation time of 50 ms, and an *m/z* range of 50–1,500. The LC-MS system was controlled using Xcalibur 2.2 SP1.48 software (Thermo Fisher Scientific), and data were collected and processed with the same software. All data that was obtained using both positive and negative ion modes were processed using the Progenesis QI data analysis software (Non-linear Dynamics, Newcastle, UK). The ranges of automatic peak picking for the C18 and HILIC assays were between 1 and 19 min and between 1 and 12 min, respectively. Next, the adduct ions of each “feature” (m/z, tR) were deconvoluted, and these features were identified in the human metabolome database (HMDB) and lipid maps.

### Metabolomic Data Analysis

The raw data were screened by correcting individual bias using QC and blank data sets. The screened data was subjected to Principal Component Analysis (PCA), Orthogonal signal correction Partial Least Square Discrimination Analysis (OPLS-DA), Variable Importance in Projection (VIP), and coefficients vs. VIP spots with the SIMCA 14.1 software program (Umetrics AB, Umea, Sweden).

### Verification of the Metabolite Profiles in the HCC Group

To verify the metabolites in the HCC group, we used an independent cohort with 72 individuals as a validation panel (Figure 1). All samples were subjected to UHPLC separation by the Thermo Scientific™ Dionex™ UltiMate™ 3000 Rapid Separation LC (RSLC) system. The gradient conditions for the C18 column and the HILIC column was the same as the above.

### Metabolite Enrichment

Metabo-Analyst version 4.0 was used for pathway enrichment analysis. The software was obtained from http://www.metaboanalyst.ca/faces/ModuleView.xhtml.

### Statistical Analysis

GraphPad Prism software (version 6.0, San Diego, California, USA) was used for statistical analysis. Continuous variables presented as mean ± standard deviation (SD). We used the Mann-Whitney *U*-test and Fisher's exact test to compare the statistical significance between different groups, respectively. The level of statistical significance was set at *p* < 0.05 two-sided for all tests. The area under the receiver-operating characteristic (ROC) curve (AUC) was calculated to evaluate the classification performance.

## Results

### Clinical Characteristics of the Subjects

Overall, 30 individuals with HBV related AFP+HCC (median 53.8 years, 25 males and 5 females), and 40 individuals with HBV related AFP–HCC (median 53.4 years, 34 males and 6 females) were recruited. Thirty HBG and 30 healthy individuals were recruited as controls. Among them, 58 individuals in the discovery panel, and 72 individuals in the validation panel were recruited (Figure 1, Table 1).

**Table 1:** Clinical characteristics of subjects belong to HCC and Control group.

**Group**	**HCC**		**Control**		p^*^
	**AFP+HCC**	**AFP–HCC**	**HBG**	**Healthy**	
**58 SUBJECTS**					
Number	15	13	16	14	
Male/female	13/2	11/2	15/1	12/2	0.701
Age	46.6 ± 3.225	43.85 ± 5.367	42.31 ± 3.979	44.79 ± 2.694	0.090
Ethnicity					0.183
Han	14	12	14	13	
Hui	1	0	2	0	
Mongols	0	1	0	1	
BCLC stage					
0	0	2			
A	1	5			
B	5	4			
C	8	2			
D	1	0			
Tumor size					
≥3 cm	9	4			
<3 cm	2	7			
Missing	4	2			
AFP	24,042 ± 33,952	3.162 ± 1.237	3.122 ± 1.183		***0.002***
WBC	6.774 ± 2.568	4.673 ± 1.576	5.154 ± 1.921		0.400
HGB	142.5 ± 14.42	139.5 ± 19.7	155.2 ± 16.51		***0.015***
PLT	150.7 ± 76.18	165.5 ± 73.62	163.3 ± 80.26		0.861
ALT	45.49 ± 21.51	40.25 ± 31.01	41.09 ± 27.22	24.49 ± 6.577	0.087
AST	53.76 ± 23.09	37.46 ± 13.02	30.13 ± 11.66	30.29 ± 5.207	***0.001***
TBIL	21.66 ± 12.2	18.57 ± 7.964	13.84 ± 4.455		***0.041***
DBIL	7.233 ± 5.688	4.846 ± 2.8	2.756 ± 1.183		***0.002***
TP	68.09 ± 6.452	65.58 ± 4.212	71.44 ± 5.831		***0.014***
ALB	38.27 ± 4.388	40.41 ± 3.763	45.04 ± 4.903		***0.000***
CREA	64.01 ± 15.33	63.52 ± 7.649	65.72 ± 20		0.252
CHOL	4.698 ± 1.642	3.813 ± 0.7259	4.319 ± 1.093		0.592
r-GT	215.7 ± 233.6	50.95 ± 35.97	45.65 ± 28.94		0.057
ALP	127.4 ± 53.16	87.18 ± 30.83	73.29 ± 19.56		***0.027***
PT	12.27 ± 1.507	12.19 ± 1.249	11.03 ± 1.229		***0.003***
PT%	89.47 ± 15.84	89.92 ± 14.44	106 ± 18.12		***0.003***
PTINR	1.094 ± 0.1297	1.086 ± 0.1067	0.9867 ± 0.1056		***0.001***
APTT	32.51 ± 5.313	33.07 ± 2.952	33.18 ± 3.34		0.804
HBV DNA >20,000 IU/mL	3/15 (20.00%)	3/13 (23.08%)	4/16 (25.00%)		0.199
**72 SUBJECTS**					
Number	15	27	14	16	
Male/female	12/3	23/4	10/4	13/3	0.561
Age	59.71 ± 6.96	57.23 ± 5.90	55.86 ± 4.99	56.4 ± 4.687	0.247
Ethnicity					0.422
Han	14	26	13	15	
Man	0	1	1	0	
Mongols	1	0	0	1	
BCLC stage					
0	0	0			
A	2	6			
B	4	11			
C	3	5			
D	6	5			
Tumor size					
≥3 cm	7	13			
<3 cm	5	14			
Missing	3	0			
AFP	2,185 ± 3,340	3.64 ± 1.761	3.202 ± 2.683		***0.003***
WBC	5.639 ± 1.978	6.089 ± 2.556	4.751 ± 1.89		0.087
HGB	138.2 ± 15.13	140.3 ± 18.49	148.4 ± 9.557		0.064
PLT	140.6 ± 69.4	151.2 ± 63.34	139.2 ± 50.4		0.892
ALT	38.56 ± 20.2	38.61 ± 23.63	47.62 ± 65.63	19.65 ± 16.07	0.439
AST	42.2 ± 16.29	34.3 ± 11.47	36.55 ± 34.39	29.91 ± 10.02	0.052
TBIL	19.2 ± 9.4	19.91 ± 11.66	15.92 ± 6.194		0.305
DBIL	5.612 ± 2.734	6.277 ± 8.084	3.257 ± 1.46		***0.023***
TP	64.24 ± 6.531	66.91 ± 5.78	69.12 ± 11.38		***0.046***
ALB	36.99 ± 4.791	40.48 ± 3.804	42.91 ± 4.838		***0.009***
CREA	67.69 ± 14.22	66.67 ± 11.82	68.98 ± 18.63		> 0.999
CHOL	3.929 ± 0.8363	3.857 ± 0.7768	4.384 ± 0.9911		0.070
r-GT	99.65 ± 100.6	68.1 ± 48.03	42.22 ± 27.4		***0.028***
ALP	88.01 ± 43.5	79.35 ± 28.15	84.6 ± 23.32		0.584
PT	12.33 ± 1.38	11.71 ± 1.19	11.52 ± 0.89		0.389
PT%	88.41 ± 16.65	95.53 ± 15.7	96.96 ± 12.16		0.475
PTINR	1.098 ± 0.1198	1.046 ± 0.1052	1.02 ± 0.08		0.261
APTT	32.28 ± 3.322	32.77 ± 5.487	33.33 ± 3.28		0.254
HBV DNA >20,000 IU/mL	2/15 (13.33%)	3/27 (11.11%)	1/14 (7.14%)		0.199

* *Compared between HCC and control groups. **Bold** and italic represent significant differences between the HCC and control groups. HCC, Hepatocellular carcinoma; BCLC, Barcelona Clinic Liver Cancer stage; AFP, alpha-fetoprotein; HBG, Hepatitis B group; WBC, White blood cell; HGB, Hemoglobin; PLT, Platelets; ALT, Alanine aminotransferase; AST, Aspartate aminotransferase; TBIL, Total bilirubin; DBIL, Direct bilirubin; TP, total protein; ALB, albumin; CREA, Creatinine; CHOL, Cholesterol; r-GT, r-glutamyl transpeptadase; ALP, Alkaline phosphatase; PT, Prothrombin time; PT%, Prothrombin time percent; PTINR, Prothrombin time international normalized ration; APTT, Activated partial thromboplatin time; HBV DNA, Hepatitis B virus deoxyribonucleic acid*.

Fifty eight individuals including AFP+HCC (*n* = 15), AFP–HCC (*n* = 13), HBG (*n* = 16), and healthy controls (*n* = 14) were analyzed for metabolic profiles (Table 1). Clinical characteristics were compared between the AFP+HCC, AFP–HCC, HBG, and healthy control groups (Table 1), and the mean age were 46.6 ± 3.225, 43.85 ± 5.367, 42.31 ± 3.979, and 44.79 ± 2.694, respectively. The level of AFP was <7 ng/ml in the AFP–HCC and HBG groups.

An independent cohort that included 72 individuals was recruited for validation (Table 1), included AFP+HCC (*n* = 15), AFP–HCC (*n* = 27), HBG (*n* = 14), and healthy controls (*n* = 16), and the mean age were 59.71 ± 6.96, 57.23 ± 5.90, 55.86 ± 4.99, and 56.4 ± 4.687, respectively. The level of AFP was <7 ng/ml in the AFP–HCC and HBG groups.

There was no significant difference found in terms of the age and gender among the HCC groups, and the control groups (including HBG and healthy groups) in those two panels (*p* > 0.05). The clinical features that included assay of direct bilirubin (DBIL), total protein (TP), and albumin (ALB) were significantly different when comparing the HCC and control groups (*p* < 0.05), in both the discovery panel and the validation panel (Table 1).

The clinical characteristics of the discovery panel and the validation panel among the AFP+HCC, AFP–HCC, HBG, and healthy groups were all compared. With the exception of age, a significantly different outcome was identified for the discovery panel and validation panel (*p* < 0.05), and most of the others that were studied were statistically non-significant (Table 2).

**Table 2:** Clinical characteristics of discovery panel and validation panel.

**Group**	**AFP+HCC**			**AFP**–**HCC**			**HBG**			**Healthy**		
	**Discovery panel**	**Validation panel**	p^*^	**Discovery panel**	**Validation panel**	p^*^	**Discovery panel**	**Validation panel**	p^*^	**Discovery panel**	**Validation panel**	p^*^
Number	15	15		13	27		16	14		14	16	
Male/female	13/2	12/3	0.852	11/2	23/4	0.948	15/1	10/4	0.313	12/2	13/3	0.854
Age	46.6 ± 3.225	59.71 ± 6.96	***0.000***	43.85 ± 5.367	57.23 ± 5.90	***0.000***	42.31 ± 3.979	55.86 ± 4.99	***0.000***	44.79 ± 2.694	56.4 ± 4.687	***0.000***
Ethnicity			1.0			0.789			0.495			0.391
Han	14	14		12	26		14	13		13	15	
Hui	1	0		0	0		2	0		0	0	
Man	0	0		0	1		0	1		0	0	
Mongols	0	1		1	0		0	0		1	1	
BCLC stage			1.000			0.147						
0	0	0		2	0							
A	1	2		5	6							
B	5	4		4	11							
C	8	3		2	5							
D	1	6		0	5							
Tumor size			1.000			0.301						
≥3 cm	9	7		4	13							
<3 cm	2	5		7	14							
Missing	4	3		2	0							
AFP	24,042 ± 33,952	2,185 ± 3,340	0.064	3.162 ± 1.237	3.64 ± 1.761	0.425	3.122 ± 1.183	3.202 ± 2.683	0.120			
WBC	6.774 ± 2.568	5.639 ± 1.978	0.202	4.673 ± 1.576	6.089 ± 2.556	***0.030***	5.154 ± 1.921	4.751 ± 1.89	0.580			
HGB	142.5 ± 14.42	138.2 ± 15.13	0.502	139.5 ± 19.7	140.3 ± 18.49	0.927	155.2 ± 16.51	148.4 ± 9.557	0.154			
PLT	150.7 ± 76.18	140.6 ± 69.4	0.794	165.5 ± 73.62	151.2 ± 63.34	0.488	163.3 ± 80.26	139.2 ± 50.4	0.473			
ALT	45.49 ± 21.51	38.56 ± 20.2	0.313	40.25 ± 31.01	38.61 ± 23.63	0.592	41.09 ± 27.22	47.62 ± 65.63	0.580	24.49 ± 6.577	19.65 ± 16.07	***0.003***
AST	53.76 ± 23.09	42.2 ± 16.29	0.230	37.46 ± 13.02	34.3 ± 11.47	0.440	30.13 ± 11.66	36.55 ± 34.39	0.984	30.29 ± 5.207	29.91 ± 10.02	0.142
TBIL	21.66 ± 12.2	19.2 ± 9.4	0.737	18.57 ± 7.964	19.91 ± 11.66	0.969	13.84 ± 4.455	15.92 ± 6.194	0.448			
DBIL	7.233 ± 5.688	5.612 ± 2.734	0.628	4.846 ± 2.8	6.277 ± 8.084	0.969	2.756 ± 1.183	3.257 ± 1.46	0.313			
TP	68.09 ± 6.452	64.24 ± 6.531	0.246	65.58 ± 4.212	66.91 ± 5.78	0.472	71.44 ± 5.831	69.12 ± 11.38	0.637			
ALB	38.27 ± 4.388	36.99 ± 4.791	0.628	40.41 ± 3.763	40.48 ± 3.804	0.927	45.04 ± 4.903	42.91 ± 4.838	0.142			
CREA	64.01 ± 15.33	67.69 ± 14.22	0.411	63.52 ± 7.649	66.67 ± 11.82	0.410	65.72 ± 20	68.98 ± 18.63	0.984			
CHOL	4.698 ± 1.642	3.929 ± 0.8363	0.350	3.813 ± 0.7259	3.857 ± 0.7768	0.648	4.319 ± 1.093	4.384 ± 0.9911	0.780			
r-GT	215.7 ± 233.6	99.65 ± 100.6	0.313	50.95 ± 35.97	68.1 ± 48.03	0.184	45.65 ± 28.94	42.22 ± 27.4	0.561			
ALP	127.4 ± 53.16	88.01 ± 43.5	0.142	87.18 ± 30.83	79.35 ± 28.15	0.865	73.29 ± 19.56	84.6 ± 23.32	0.354			
PT	12.27 ± 1.507	12.33 ± 1.38	0.766	12.19 ± 1.249	11.71 ± 1.19	0.300	11.03 ± 1.229	11.52 ± 0.89	0.384			
PT%	89.47 ± 15.84	88.41 ± 16.65	0.766	89.92 ± 14.44	95.53 ± 15.7	0.366	106 ± 18.12	96.96 ± 12.16	0.650			
PTINR	1.094 ± 0.1297	1.098 ± 0.1198	0.737	1.086 ± 0.1067	1.046 ± 0.1052	0.313	0.9867 ± 0.1056	1.02 ± 0.08	0.384			
APTT	32.51 ± 5.313	32.28 ± 3.322	0.576	33.07 ± 2.952	32.77 ± 5.487	0.456	33.18 ± 3.34	33.33 ± 3.28	0.837			
HBV DNA >20,000 IU/mL	3/15 (20.00%)	2/15 (13.33%)	0.710	3/13 (23.08%)	3/27 (11.11%)	0.505	4/16 (25.00%)	1/14 (7.14%)	0.423			

* *Compared between discovery panel and validation panel. **Bold** and italic represent significant difference between discovery panel and validation panel. HCC, Hepatocellular carcinoma; BCLC, Barcelona Clinic Liver Cancer stage; AFP, alpha-fetoprotein; HBG, Hepatitis B group; WBC, White blood cell; HGB, Hemoglobin; PLT, Platelets; ALT, Alanine aminotransferase; AST, Aspartate aminotransferase; TBIL, Total bilirubin; DBIL, Direct bilirubin; TP, total protein; ALB, albumin; CREA, Creatinine; CHOL, Cholesterol; r-GT, r-glutamyl transpeptadase; ALP, Alkaline phosphatase; PT, Prothrombin time; PT%, Prothrombin time percent; PTINR, Prothrombin time international normalized ration; APTT, Activated partial thromboplatin time; HBV DNA, Hepatitis B virus deoxyribonucleic acid*.

### PCA and OPLS-DA

**Figure 2:**
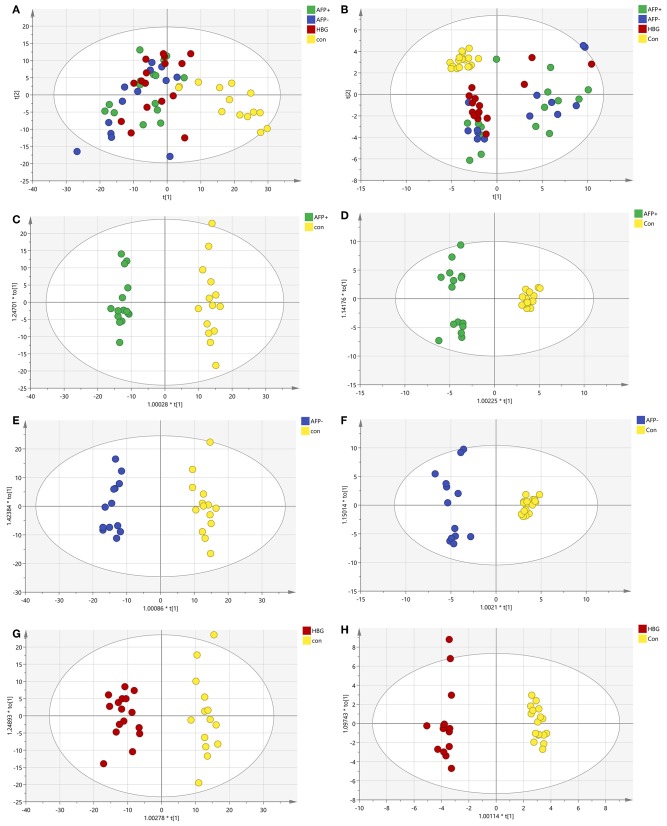
PCA and OPLS-DA of the metabolic profiles of the plasma samples in the AFP+HCC, AFP-HCC, HBG, and healthy groups. **(A)** PCA analysis in C18; **(B)** PCA analysis in HILIC; **(C)** OPLS-DA analysis in C18 for AFP+ and Con; **(D)** OPLS-DA analysis in the HILIC for AFP+ and Con; **(E)** OPLS-DA analysis in C18 for AFP- and Con; **(F)** OPLS-DA analysis in the HILIC for AFP- and Con; **(G)** OPLS-DA analysis in C18 for HBG and Con; **(H)** OPLS-DA analysis in the HILIC for HBG and Con. Green, blue, red, and yellow nodes, respectively, represented subject samples in the AFP+HCC, AFP-HCC, HBG, and healthy groups. PCA, principal component analysis; OPLS-DA, orthogonal partial least-squares-discriminant analysis; HILIC, hydrophilic interaction liquid chromatography; AFP, alpha-fetoproteins; HCC, hepatocellular carcinoma; HBG, hepatitis B group.

Liquid chromatography was applied to separate lipid and polarized components from the plasma samples. The metabolic profiles of the plasma samples in the AFP+HCC, AFP–HCC, HBG, and healthy groups were displayed by PCA (Figures 2A,B). PCA analysis suggested distinct clusters of samples from different groups in both the C18 column (R2X = 0.481, Q2 = 0.392; Figure 2A) and the HILIC columns (R2X = 0.468, Q2 = 0.272; Figure 2B). OPLS-DA was further used to analyze the metabolic differences among the AFP+HCC with healthy groups (Figure 2C for C18 column, Figure 2D for HILIC columns), AFP–HCC with healthy groups (Figure 2E for C18 column, Figure 2F for HILIC columns), HBG with healthy groups (Figure 2G for C18 column, Figure 2H for HILIC columns). All OPLS-DA models between different groups indicated adequate classification.

### Metabolic Profiles in Different Groups Compared With the Healthy Group

**Figure 3:**
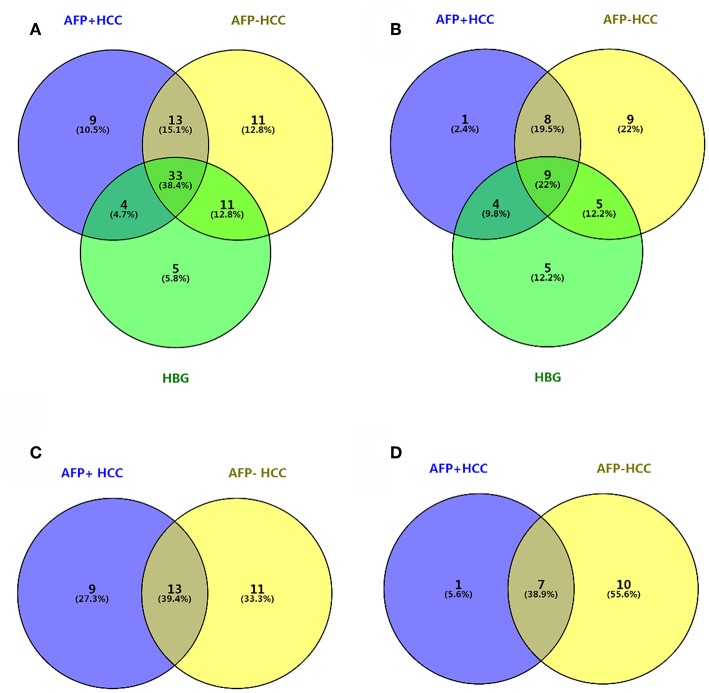
Venn diagram between AFP+HCC, AFP–HCC, HBG, and the healthy groups. **(A)** Results of C18 as compared with healthy group; **(B)** results of HILIC as compared with healthy group; **(C)** results of C18 as compared with HBG group; **(D)** results of HILIC as compared with HBG group.

Significantly changed metabolites (SCM; defined by based VIP > 1 and *p* < 0.05) were selected for subsequent chemical structure identification. The metabolic profiles of the AFP+HCC, AFP–HCC, HBG were substantially different from that of the healthy control group, both in the C18 column (Figure 3A and Table S2) and the HILIC column (Figure 3B and Table S3). In addition, a substantial difference was observed between the AFP+HCC, AFP–HCC, and HBG groups both in the C18 column (Table S2) and the HILIC column (Table S3).

### Metabolic Profiles in the HCC Group as Compared With HBG

**Figure 4:**
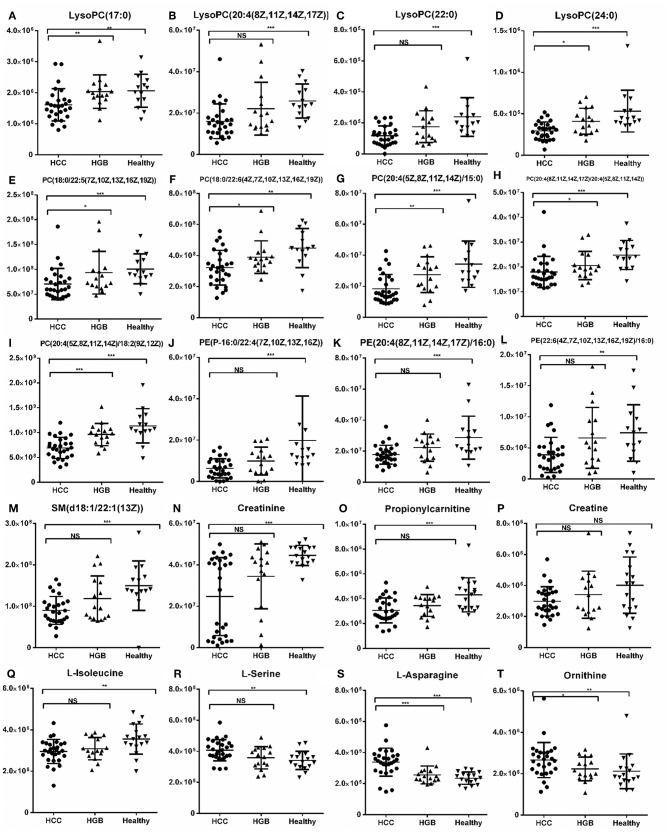
Histogram showing the abundance of SCMs generated from C18 and HILIC in the HCC, HBG, and healthy groups of the discovery phase. **(A)** LysoPC (17:0); **(B)** LysoPC (20:4(18Z, 11Z,14Z,17Z); **(C)** LysoPC (22:0); **(D)** LysoPC (24:0); **(E)** PC (18:0/22:5(7Z,10Z,13Z,16Z,19Z)); **(F)** PC (18:0/22:5(7Z,10Z,13Z,16Z,19Z)); **(G)** PC (20:4(5Z,8Z,11Z,14Z)/15:0); **(H)** PC (20:4(8Z,11Z,14Z,17Z)/20:4(5Z,8Z,11Z,14Z)); **(I)** PC (20:4(5Z,8Z,11Z,14Z)/18:2(9Z,12Z)); **(J)** PE (P-16:0/22:4 (7Z,10Z,13Z,16Z)); **(K)** PE (20:4(8Z,11Z,14Z,17Z)/16:0); **(L)** PE (22:6(4Z,7Z,10Z,13Z,16Z,19Z)/16:0); **(M)** SM (d18:1/22:1(13Z)); **(N)** Creatinine; **(O)** Propionylcarnitine; **(P)** Creatine; **(Q)** L-Isoleucine; **(R)** L-Serine; **(S)** L-Asparagine; **(T)** Ornithine. ^*^indicates *p* < 0.05; ^**^indicates *p* < 0.01; and ^***^indicates *p* < 0.001. Value of *p* < 0.05 indicates statistical significance. SCMs, significantly changed metabolites; NS, non-statistical significance. The HCC, HBG, and healthy groups in the discovery phase included 28, 16, and 14 subjects, respectively.

We then selected common metabolites in the C18 column (Figure 3C) and the HILIC column (Figure 3D) from the AFP+HCC and the AFP–HCC groups, respectively, and as compared with HBG. There were 13 SCMs for the C18 column (Table S4) and 7 SCMs for the HILIC column (Table S5) identified for both the AFP+HCC and the AFP–HCC groups. In addition, there were 13 SCMs for the C18 column and 6 out of 7 SCMs for the HILIC column (with the exception of creatine) with significant difference when compared HCC with healthy control groups (*p* < 0.05; Figure 4). Otherwise, comparing HCC group with HBG group, we found 7 out of 13 SCMs for C18 column (LysoPC (24:0), LysoPC (17:0), PC (20:4(8Z,11Z,14Z,17Z)/20:4(5Z,8Z,11Z,14Z)),PC (20:4(5Z,8Z,11Z,14Z)/18:2(9Z,12Z)),PC (18:0/22:5(7Z,10Z,13Z,16Z,19Z)),PC (20:4(5Z,8Z,11Z,14Z)/15:0), PC (18:0/22:6(4Z,7Z,10Z,13Z,16Z,19Z))), and 2 out of 7 SCMs for HILIC (L-asparagine and ornithine) with significant difference (*p* < 0.05; Figure 4). Moreover, there were no significant difference between the AFP+HCC and the AFP–HCC groups for these SCMs (13 SCMs for the C18 column and 7 SCMs for the HILIC column, data not shown).

### Validation of the Potential Metabolic Biomarkers

**Figure 5:**
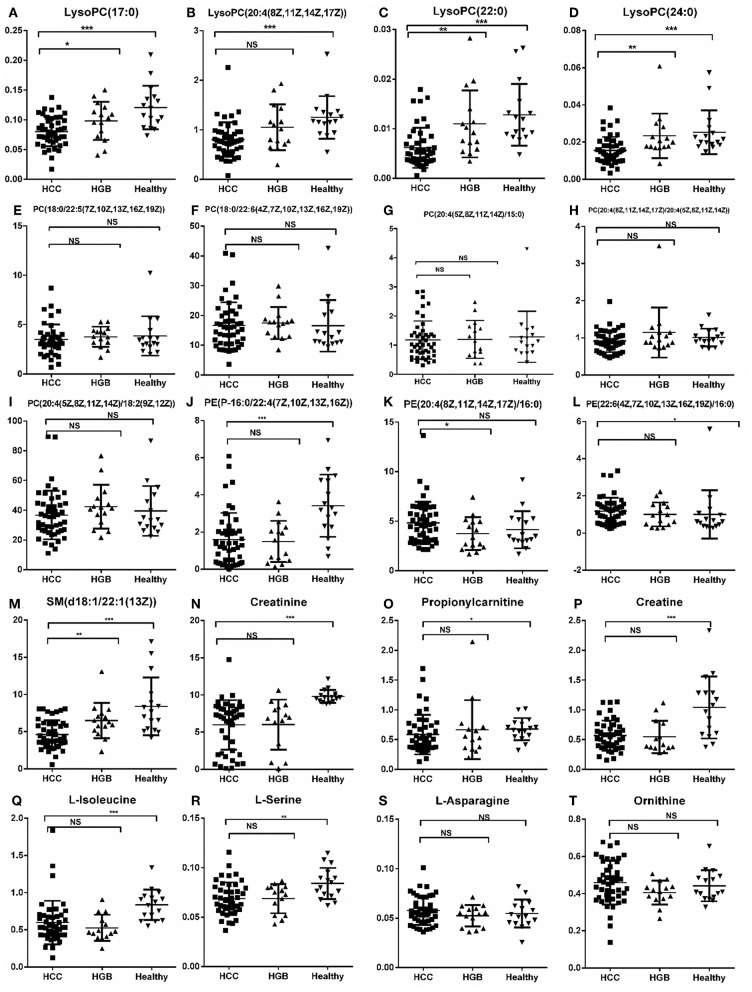
Relative abundance of SCMs generated from the C18 column and the HILIC column in HCC, HBG, and healthy groups of 72 subjects. **(A)** LysoPC (17:0); **(B)** LysoPC (20:4(18Z, 11Z,14Z,17Z); **(C)** LysoPC (22:0); **(D)** LysoPC (24:0); **(E)** PC (18:0/22:5(7Z,10Z,13Z,16Z,19Z)); **(F)** PC (18:0/22:5(7Z,10Z,13Z,16Z,19Z)); **(G)** PC (20:4(5Z,8Z,11Z,14Z)/15:0); **(H)** PC (20:4(8Z,11Z,14Z,17Z)/20:4(5Z,8Z,11Z,14Z)); **(I)** PC (20:4(5Z,8Z,11Z,14Z)/18:2(9Z,12Z)); **(J)** PE (P-16:0/22:4(7Z,10Z,13Z,16Z)); **(K)** PE (20:4(8Z,11Z,14Z,17Z)/16:0); **(L)** PE (22:6(4Z,7Z,10Z,13Z,16Z,19Z)/16:0); **(M)** SM (d18:1/22:1(13Z)); **(N)** Creatinine; **(O)** Propionylcarnitine; **(P)** Creatine; **(Q)** L-Isoleucine; **(R)** L-Serine; **(S)** L-Asparagine; **(T)** Ornithine. ^*^indicates *p* < 0.05, ^**^indicates *p* < 0.01, and ^***^indicates *p* < 0.001. An value of *p* < 0.05 indicates statistical significance. SCMs, significantly changed metabolites; NS, non-statistical significance. Targeted metabolomic analysis was used to validate the abundance of SCMs in 72 subjects, including HCC, HBG, and healthy groups included 42, 14, and 16 subjects, respectively.

We validated these metabolites (13 SCMs for the C18 column and 7 SCMs for the HILIC column) in a validation panel. Targeted metabolomic analysis was performed and the concentration of SCMs was measured. Compared with the healthy control group, 7 SCMs for the C18 column and 5 SCMs for the HILIC column were identified (*p* < 0.05; Figure 5). In addition, when compared with HBG group, there were 5 SCMs for the C18 column and none SCMs for the HILIC column were identified (*p* < 0.05; Figure 5). Moreover, there were also no significant difference when comparing the AFP+HCC group and the AFP–HCC group for these SCMs (13 SCMs for the C18 column and 7 SCMs for the HILIC column, data not shown).

Comparing the tendency of these metabolites (13 SCMs for the C18 and 7 SCMs for the HILIC columns) in the training and validation panels, we found that 10 SCMs had the same metabolic tendency in both panels, and all of them were significantly decreased in the HCC group as compared with the healthy controls. Then, compared with the HBG group, only LysoPC (24:0) was significantly decreased in the HCC groups for both panels.

**Figure 6:**
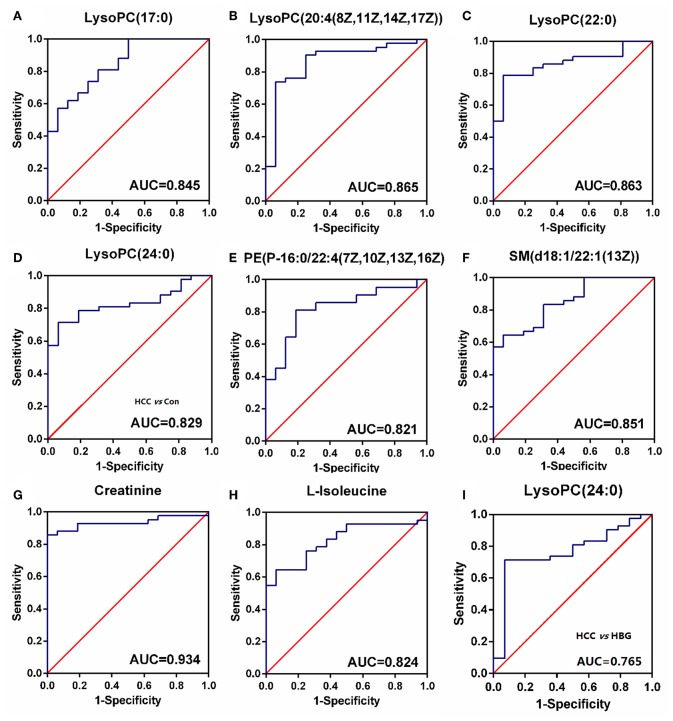
The ROC curve. **(A–H)** The ROC curve for SCMs between HCC subjects and healthy group. **(A)** LysoPC (22:0); **(B)** LysoPC (24:0); **(C)** LysoPC (17:0); **(D)** LysoPC (20:4 (8Z,11Z,14Z,17Z)); **(E)** SM (d18:1/22:1(13Z)); **(F)** PE (P-16:0/22:4 (7Z, 10Z, 13Z, and 16Z)); **(G)** L-Isoleucine; **(H)** Creatinine. The HCC and healthy groups included 42, and 16 subjects, respectively. **(I)** The ROC curve for LysoPC (24:0) between HCC subjects and HBG. The HCC and HBG included 42, and 14 subjects, respectively. ROC, receiver operating characteristic curve. ROC curves and AUC were obtained by GraphPad software version 6.0.

ROC analyses, which calculate the area under the curve (AUC), were applied for 10 SCMs. Six SCMs for the C18 column, including LysoPC (17:0) (Figure 6A), LysoPC (20:4(8Z,11Z,14Z,17Z)) (Figure 6B), LysoPC (22:0) (Figure 6C), LysoPC (24:0) (Figure 6D), PE (P-16:0/22:4(7Z,10Z,13Z,16Z)) (Figure 6E), SM (d18:1/22:1(13Z)) (Figure 6F) had a diagnostic value in distinguishing the HCC group from the healthy group. The AUCs were 0.845, 0.865,0.863, 0.829, 0.821, and 0.851, respectively. Two SCMs for the HILIC column including creatinine (Figure 6G), and L-isoleucine (Figure 6H) had diagnostic value in distinguishing the HCC group from the healthy group, with AUC values of 0.934 and 0.824, respectively. Finally, to detect the diagnostic value of LysoPC (24:0) (Figure 6I) between the HCC and HBG groups, the ROC analyses showed that the AUC was 0.765. And none of the SCMs for the HILIC column had diagnostic value in distinguishing HCC from HBG.

### The Metabolic Pathway of HCC

**Figure 7:**
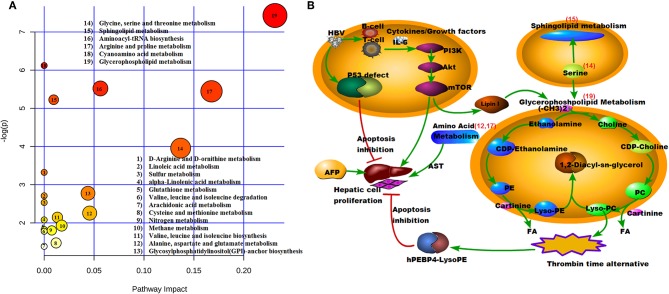
Model illustrating metabolic processes. **(A)** Pathway model. X = pathway impact, y = –log (p). 19, Glycerophospholipid metabolism; 17, Arginine and proline metabolism; 14, Glycine, serine, and threonine metabolism; **(B)** Signaling pathways. The number of red is consistent with **(A)**. 19, Glycerophospholipid metabolism; 17, Arginine and proline metabolism; 15, Sphingolipid metabolism; 14, Glycine, serine, and threonine metabolism; 12, Alanine, aspartate, and glutamate metabolism.

MetaboAnalyst version 4.0 was used to identity the related pathways of those SCMs. Three metabolic pathways (with larger circles in the Figure 7A) were significantly enriched, including Glycerophospholipid metabolism (circle 19), Arginine and proline metabolism (circle 17), and Glycine, serine, and threonine metabolism (circle 14). Six of eight SCMs were correlated with lipid metabolism, including LysoPC (22:0), LysoPC (24:0), LysoPC (17:0), LysoPC (20:4(8Z,11Z,14Z,17Z)), PE (P-16:0/22:4(7Z,10Z,13Z,16Z)), and SM (d18:1/22:1(13 Z)). Additionally, all of them were significantly decreased in the HCC group (Figure 5).

To better understand the mechanism of these SCMs, the reported signaling pathways were involved in cell growth, cell proliferation, and cell cycle in HCC and the potential metabolic signaling pathways that were identified in our work were schematically depicted in Figure 7B. Importantly, those identified signaling pathways indicated metabolic dysregulation during the process of HCC tumorigenesis.

## Discussion

In order to improve the sensitivity and specificity of diagnostic relevant metabolomic biomarkers for HCC patients, we collected plasma samples from HBV infected HCC. This included data sets for gender- and age-matched AFP–HCC and AFP+HCC subjects, as well as HCC groups, and HBG and healthy individuals as control groups, so that we could perform a comprehensive metabolomic profiling analysis. In results we found 20 common SCMs in both the AFP–HCC and the AFP+HCC groups. Considering that metabolomics analyses might be affected by age, we divided the discovery panel and validation panel to individuals that were aged 40–50 and 50–70 years, respectively. Then, we further validated these 20 SCMs in 72 subjects, and found that there were 10 SCMs displaying the same tendency in both the discovery panel and the validation panel.

Next, we analyzed the diagnostic value of the markers among HCC, HBG, and healthy groups. The ROC curve analyses indicated that 8 SCMs had a high discriminatory ability when comparing HCC and healthy controls, indicating that they might serve as potential biomarkers. Compared to HBG, the abundance of LysoPC (24:0) in the HCC group was significantly decreased in both the discovery and validation panel. The AUC value for LysoPC (24:0) reached a value of 0.765, which suggested that it can discriminate HCC and HBG individuals (Figure 6I).

The functional cluster analysis showed that 6 out of 8 SCMs, including LysoPC (17:0) (Figure 6A), LysoPC (20:4(8Z,11Z,14Z,17Z)) (Figure 6B), LysoPC (22:0) (Figure 6C), LysoPC (24:0) (Figure 6D), PE (P-16:0/22:4(7Z,10Z,13Z,16Z)) (Figure 6E), and SM (d18:1/22:1(13Z)) (Figure 6F), whose molecular formulas are C25H52NO7P, C28H50NO7P, C30H62NO7P, C32H66NO7P, C43H78NO7P, and C45H89N2O6P, were correlated with lipid metabolism. Among them, LysoPC (22:0) belongs to members of the lysophosphatidylcholines (LPCs), which are formed by hydrolysis of phosphatidylcholines (PCs) by the enzyme phospholipase A2 (16). The other SCMs were PE (P-16:0/22:4(7Z, 10Z, 13Z, 16Z)) and SM (d18:1/22:1(13Z)), which are essential components of membrane structure, signal transduction and lipoprotein metabolism.

L-isoleucine has a molecular formula of C6H13NO2 and is an essential amino acid that is involved in protein synthesis in the human body, and is an aromatic amino acid. The metabolites included L-isoleucine, and provided a diagnostic model that could discriminate HCC and normal control very well (17).

The pathway analysis showed that the different molecules played important roles in activated tumor cell function and tumorigenesis, and included protein and lipid metabolism, and especially sphingolipid metabolism (18). Our ROC analysis showed that lipid metabolism was significantly different among the HCC, HBG, and healthy control groups. Prior studies considered that the development of HCC can cause a hypoxic microenvironment; thus, tumor cells need lipid metabolism to change the metabolic mode and adapt to the change (19, 20). Our observations further support this notion, and increased cellular lipid metabolism and increased consumption resulted in decreased expression levels of lipid metabolite in the plasma. According to the metabolic character of the lipid, activation of lipid metabolism in cells, and especially that of LysoPE and LysoPC, can promote synthesis of human phosphatidyl-ethanolamine-binding protein 4 (hPEBP4), which is an anti-apoptotic protein, and can associate with HBV to activate the PI3K/Akt/mTOR signaling pathway to promote tumorigenesis, development, and metastasis (21–23).

Nutritional and functional decline (cachexia) is a common phenomenon seen in cancer patients, and even the loss of skeletal muscle is tightly connected to poor performance status, and poor response to treatment and a poor prognostic outcome for those patients (24, 25). In our results, creatinine and creatine in HCC were down-regulated as compared with the healthy control group. In addition, it has been reported that it might imply the loss of muscle in HCC patients, which might then contribute to the observed high morbidity and mortality seen in HCC (24).

As the main pathogenic agent of HCC genesis, many studies have found that AFP can mediate HBx protein activation, and promote the development of HCC (26), with an accompanied suppression of host protective immunity (27). Recently, research studies have found that AFP high and AFP low subjects display differential proteomic profiles, and that the poor prognosis seen in HCC is associated with cholesterol homeostasis (28). According to the clinical data and pathway analysis, our results also showed that HBV DNA levels were higher in the AFP+HCC group than that found in the AFP–HCC group. In addition, both HBV and AFP can biologically collaborate to promote HCC development. At the same time, AFP possesses a variety of biological functions. The clinical data showed that the level of DBIL in the AFP+HCC group was higher than that found in the AFP–HCC group (Table 1, *p* < 0.05). According to the above analysis, different AFP expression levels might have a differential metabolic profile.

In this study, we found some biomarkers might have utility in diagnosing HBV-associated HCC. Several studies have also proposed biomarkers for each HCC stage, from prevention to progression (29). And in our results, we also found that some metabolomic biomarkers were correlated with tumor stage or tumor size (data not shown). Thus, future studies will strive to investigate those biomarkers in larger subject cohorts, including different stages of HCC. We will also seek to explore the different metabolic biomarkers that might distinguish AFP–HCC and AFP+HCC.

The employment of more than one biomarker could provide a dynamic and powerful approach to advance our understanding of the spectrum of HCC, with diverse applications in clinical epidemiology, laboratory screening, and physician confirmed diagnosis and prognosis. Our results indicated that fatty acid and lipid metabolism might promote HCC genesis, including the interplay of glycerophospholipid metabolism and some select amino acid metabolic activity; however, these ideas require formal confirmation. On the other hand, the limitation of this study, is that there were several factors, which included environmental or behavioral factors (e.g., diet) that might have formally played an important role in the concentrations of metabolic products in an individual patient (30)—a consideration that was not further explored in this particular study.

## Data Availability Statement

The raw data supporting the conclusions of this manuscript will be made available by the authors, without undue reservation, to any qualified researcher.

## Ethics Statement

The studies involving human participants were reviewed and approved by the local ethics committee of Beijing You'an hospital, of the Capital Medical University. The patients/participants provided their written informed consent to participate in this study.

## Author Contributions

JS, TZ, and YonghongZ carried out the studies, participated in collecting data, and drafted the manuscript. YananZ performed the statistical analysis. LQ participated in its design. KL, HS, and YanZ helped to draft the manuscript. All authors read and approved the final manuscript.

### Conflict of Interest

The authors declare that the research was conducted in the absence of any commercial or financial relationships that could be construed as a potential conflict of interest.
