# Supplementary material for: Metabolomic Profiles for HBV Related Hepatocellular Carcinoma Including Alpha-Fetoproteins Positive and Negative Subtypes
Source: Front Oncol. 2019 Oct 15;9:1069. doi: 10.3389/fonc.2019.01069 (PMC6803550; doi:10.3389/fonc.2019.01069)
Supplement: Supplementary file 1 [file Table_1.DOCX]

**Table S1.The gradient conditions for C18 and HILIC**

|  | Time (min) | A (v %) | B (v %) |
| --- | --- | --- | --- |
| **C18** | 0 | 80 | 20 |
|  | 2 | 70 | 30 |
|  | 5 | 55 | 45 |
|  | 6.5 | 40 | 60 |
|  | 12 | 35 | 65 |
|  | 14 | 15 | 85 |
|  | 17.5 | 0 | 100 |
|  | 18 | 0 | 100 |
|  | 18.1 | 80 | 20 |
|  | 19.5 | 80 | 20 |
|  |  |  |  |
| **HILIC** | 0 | 95 | 5 |
|  | 1 | 95 | 5 |
|  | 7 | 50 | 50 |
|  | 9 | 50 | 50 |
|  | 9.1 | 95 | 5 |
|  | 13 | 95 | 5 |
