# Supplementary material for: Metabolomic Profiles for HBV Related Hepatocellular Carcinoma Including Alpha-Fetoproteins Positive and Negative Subtypes
Source: Front Oncol. 2019 Oct 15;9:1069. doi: 10.3389/fonc.2019.01069 (PMC6803550; doi:10.3389/fonc.2019.01069)
Supplement: Supplementary file 2 [file Table_2.DOCX]

**Table S2. The SCMs of C18 column chromatography in the AFP+HCC, AFP-HCC, and HGB groups as compared the healthy group**

| AFP+ | AFP- | HBG |
| --- | --- | --- |
| PC(20:4(5Z,8Z,11Z,14Z)/18:2(9Z,12Z)) | PC(18:3(9Z,12Z,15Z)/16:0) | PC(18:3(9Z,12Z,15Z)/16:0) |
| PC(20:4(5Z,8Z,11Z,14Z)/20:5(5Z,8Z,11Z,14Z,17Z)) | PC(18:2(9Z,12Z)/15:0) | SM(d18:0/16:1(9Z)) |
| Cer(d18:0/16:0) | SM(d18:0/16:1(9Z)) | PC(20:4(5Z,8Z,11Z,14Z)/20:5(5Z,8Z,11Z,14Z,17Z)) |
| SM(d18:1/18:1(11Z)) | PC(20:4(5Z,8Z,11Z,14Z)/18:2(9Z,12Z)) | Cer(d18:0/16:0) |
| PC(16:0/20:4(5Z,8Z,11Z,14Z)) | PC(20:4(5Z,8Z,11Z,14Z)/20:5(5Z,8Z,11Z,14Z,17Z)) | SM(d18:1/18:1(11Z)) |
| PE(22:6(4Z,7Z,10Z,13Z,16Z,19Z)/16:0) | Cer(d18:0/16:0) | PC(16:0/20:4(5Z,8Z,11Z,14Z)) |
| PC(18:2(9Z,12Z)/16:0) | PC(16:0/16:1(9Z)) | PC(18:2(9Z,12Z)/16:0) |
| PC(20:4(5Z,8Z,11Z,14Z)/16:1(9Z)) | SM(d18:1/18:1(11Z)) | PC(20:4(5Z,8Z,11Z,14Z)/16:1(9Z)) |
| PE(20:4(8Z,11Z,14Z,17Z)/16:0) | PC(16:0/20:4(5Z,8Z,11Z,14Z)) | PE(18:2(9Z,12Z)/16:0) |
| PC(20:4(8Z,11Z,14Z,17Z)/20:4(5Z,8Z,11Z,14Z)) | PE(22:6(4Z,7Z,10Z,13Z,16Z,19Z)/16:0) | PC(P-16:0/20:4(8Z,11Z,14Z,17Z)) |
| PE(18:2(9Z,12Z)/16:0) | PC(18:2(9Z,12Z)/16:0) | PE(18:2(9Z,12Z)/18:1(9Z)) |
| PC(P-16:0/20:4(8Z,11Z,14Z,17Z)) | PC(20:4(5Z,8Z,11Z,14Z)/16:1(9Z)) | PC(o-16:0/16:1(9Z)) |
| PE(18:2(9Z,12Z)/18:1(9Z)) | PE(20:4(8Z,11Z,14Z,17Z)/16:0) | PC(o-16:0/20:4(8Z,11Z,14Z,17Z)) |
| PC(o-16:1(9Z)/18:2(9Z,12Z)) | PC(20:4(8Z,11Z,14Z,17Z)/20:4(5Z,8Z,11Z,14Z)) | PC(o-16:1(9Z)/18:2(9Z,12Z)) |
| PC(18:0/22:6(4Z,7Z,10Z,13Z,16Z,19Z)) | PC(15:0/18:1(11Z)) | SM(d18:0/18:1(11Z)) |
| PC(20:4(8Z,11Z,14Z,17Z)/18:0) | PE(18:2(9Z,12Z)/16:0) | PC(16:0/18:1(11Z)) |
| PC(18:0/22:5(7Z,10Z,13Z,16Z,19Z)) | PC(P-16:0/20:4(8Z,11Z,14Z,17Z)) | PC(20:4(8Z,11Z,14Z,17Z)/18:0) |
| SM(d18:0/18:0) | PE(18:2(9Z,12Z)/18:1(9Z)) | PC(18:0/18:2(9Z,12Z)) |
| PC(18:0/18:2(9Z,12Z)) | PC(o-16:0/20:4(8Z,11Z,14Z,17Z)) | PC(P-16:0/18:1(11Z)) |
| PC(20:4(5Z,8Z,11Z,14Z)/15:0) | PC(o-16:1(9Z)/18:2(9Z,12Z)) | PC(18:0/20:3(8Z,11Z,14Z)) |
| PC(P-16:0/18:1(11Z)) | PC(P-18:0/20:4(5Z,8Z,11Z,14Z)) | PE(18:2(9Z,12Z)/18:0) |
| PE(18:2(9Z,12Z)/18:0) | SM(d18:0/18:1(11Z)) | PC(18:0/22:4(7Z,10Z,13Z,16Z)) |
| PC(18:0/22:4(7Z,10Z,13Z,16Z)) | PC(18:0/22:6(4Z,7Z,10Z,13Z,16Z,19Z)) | SM(d18:1/20:0) |
| PE(P-16:0/22:4(7Z,10Z,13Z,16Z)) | PC(O-16:0/18:2(9Z,12Z)) | PE(P-18:0/18:2(9Z,12Z)) |
| SM(d18:1/22:1(13Z)) | PC(16:0/18:1(11Z)) | PE(18:0/18:1(11Z)) |
| PE(P-18:0/18:2(9Z,12Z)) | PC(20:4(8Z,11Z,14Z,17Z)/18:0) | SM(d18:0/22:1(13Z)) |
| PE(18:0/18:1(11Z)) | PC(18:0/22:5(7Z,10Z,13Z,16Z,19Z)) | SM(d18:1/24:1(15Z)) |
| PC(o-16:0/18:0) | PC(18:0/18:2(9Z,12Z)) | SM(d18:0/22:0) |
| SM(d18:0/22:1(13Z)) | PC(20:4(5Z,8Z,11Z,14Z)/15:0) | SM(d18:0/24:1(15Z)) |
| SM(d18:1/24:1(15Z)) | PC(P-16:0/18:1(11Z)) | Cer(d18:1/24:1(15Z)) |
| SM(d18:0/26:1(17Z)) | PC(18:0/20:3(8Z,11Z,14Z)) | Ceramide (d18:1/22:0) |
| SM(d18:0/24:1(15Z)) | PE(18:2(9Z,12Z)/18:0) | Cer(d18:1/23:0) |
| Cer(d18:1/24:1(15Z)) | PC(18:0/22:4(7Z,10Z,13Z,16Z)) | Ceramide (d18:1/24:0) |
| Ceramide (d18:1/22:0) | SM(d18:1/20:0) | Ceramide (d18:1/25:0) |
| Cer(d18:1/23:0) | PE(P-16:0/22:4(7Z,10Z,13Z,16Z)) | LysoPC(14:0) |
| Ceramide (d18:1/24:0) | PC(18:0/18:1(11Z)) | LysoPC(16:1(9Z)) |
| Ceramide (d18:1/25:0) | SM(d18:1/22:1(13Z)) | LysoPE(0:0/20:4(8Z,11Z,14Z,17Z)) |
| LysoPC(14:0) | PC(18:0/20:2(11Z,14Z)) | LysoPC(18:2(9Z,12Z)) |
| LysoPC(22:6(4Z,7Z,10Z,13Z,16Z,19Z)) | PE(P-18:0/18:2(9Z,12Z)) | LysoPE(18:2(9Z,12Z)/0:0) |
| LysoPE(22:6(4Z,7Z,10Z,13Z,16Z,19Z)/0:0) | PE(18:0/18:1(11Z)) | LysoPC(16:0) |
| LysoPC(20:4(8Z,11Z,14Z,17Z)) | SM(d18:0/22:1(13Z)) | LysoPE(0:0/16:0) |
| LysoPC(18:2(9Z,12Z)) | SM(d18:1/24:1(15Z)) | LysoPE(0:0/18:1(11Z)) |
| LysoPE(20:4(5Z,8Z,11Z,14Z)/0:0) | SM(d18:0/22:0) | LysoPE(0:0/22:4(7Z,10Z,13Z,16Z)) |
| LysoPE(18:2(9Z,12Z)/0:0) | SM(d18:1/23:0) | LysoPC(P-16:0) |
| LysoPC(20:3(5Z,8Z,11Z)) | SM(d18:0/24:1(15Z)) | LysoPC(20:2(11Z,14Z)) |
| LysoPC(16:0) | Cer(d18:1/24:1(15Z)) | LysoPC(18:0) |
| LysoPE(0:0/16:0) | Ceramide (d18:1/22:0) | LysoPE(18:0/0:0) |
| LysoPC(22:4(7Z,10Z,13Z,16Z)) | Cer(d18:1/23:0) | LysoPC(20:1(11Z)) |
| LysoPE(18:1(11Z)/0:0) | Ceramide (d18:1/24:0) | LysoPC(20:0) |
| LysoPC(P-16:0) | Cer(d18:0/24:0) | SM(d18:0/16:1(9Z)(OH)) |
| LysoPC(20:2(11Z,14Z)) | LysoPC(14:0) | Cer(d18:0/14:0) |
| LysoPC(17:0) | LysoPC(16:1(9Z)) | SM(d18:0/14:0) |
| LysoPC(18:0) | LysoPC(20:4(8Z,11Z,14Z,17Z)) | PC(14:0/18:2(9Z,12Z)) |
| LysoPE(18:0/0:0) | LysoPC(16:0) |  |
| LysoPC(20:0) | LysoPE(0:0/16:0) |  |
| LysoPC(22:0) | LysoPC(P-16:0) |  |
| Cer(d18:0/14:0) | LysoPC(17:0) |  |
| LysoPC(24:0) | LysoPC(18:0) |  |
| PC(14:0/18:2(9Z,12Z)) | LysoPE(18:0/0:0) |  |
|  | LysoPC(20:0) |  |
|  | LysoPC(22:0) |  |
|  | SM(d18:0/16:1(9Z)(OH)) |  |
|  | Cer(d18:0/14:0) |  |
|  | LysoPC(24:0) |  |
|  | SM(d18:1/14:0) |  |
|  | SM(d18:0/14:0) |  |
|  | PC(14:0/18:2(9Z,12Z)) |  |
|  | PC(20:4(5Z,8Z,11Z,14Z)/18:4(6Z,9Z,12Z,15Z)) |  |

**Key:** green: the specific C18 of the AFP plus HCC or AFP-HCC groups as compared the HGB group
