# Supplementary material for: Metabolomic Profiles for HBV Related Hepatocellular Carcinoma Including Alpha-Fetoproteins Positive and Negative Subtypes
Source: Front Oncol. 2019 Oct 15;9:1069. doi: 10.3389/fonc.2019.01069 (PMC6803550; doi:10.3389/fonc.2019.01069)
Supplement: Supplementary file 3 [file Table_3.DOCX]

**Table S3. The SCMs of HILIC column chromatography in AFP+HCC, AFP-HCC, and HGB groups as compared the healthy group**

| AFP+ | AFP- | HBG |
| --- | --- | --- |
| Stearoylcarnitine | L-Palmitoylcarnitine | Niacinamide |
| Vaccenyl carnitine | Alpha-linolenyl carnitine | Stearoylcarnitine |
| L-Palmitoylcarnitine | Linoelaidyl carnitine | Vaccenyl carnitine |
| Alpha-linolenyl carnitine | trans-Hexadec-2-enoyl carnitine | L-Palmitoylcarnitine |
| Linoelaidyl carnitine | Tetradecanoylcarnitine | Alpha-linolenyl carnitine |
| trans-Hexadec-2-enoyl carnitine | trans-2-Tetradecenoylcarnitine | Linoelaidyl carnitine |
| Tetradecanoylcarnitine | Dodecanoylcarnitine | trans-Hexadec-2-enoyl carnitine |
| Propionylcarnitine | trans-2-Dodecenoylcarnitine | Tetradecanoylcarnitine |
| L-Acetylcarnitine | 5'-Methylthioadenosine | trans-2-Tetradecenoylcarnitine |
| Creatinine | L-Octanoylcarnitine | Dodecanoylcarnitine |
| L-Isoleucine | Butyrylcarnitine | trans-2-Dodecenoylcarnitine |
| L-Carnitine | Propionylcarnitine | 5'-Methylthioadenosine |
| L-Leucine | L-Acetylcarnitine | Decanoylcarnitine |
| Betaine | Creatinine | L-Octanoylcarnitine |
| L-Proline | L-Isoleucine | L-Hexanoylcarnitine |
| Creatine | L-Pipecolic acid | L-Acetylcarnitine |
| L-Alanine | L-Tryptophan | L-Leucine |
| L-Serine | L-Leucine | L-Methionine |
| L-Asparagine | Betaine | L-Proline |
| Symmetric dimethylarginine | 1-Methyladenosine | L-Alanine |
| L-Arginine | Creatine | Symmetric dimethylarginine |
| Ornithine | L-Alanine | L-Targinine |
|  | 4-Hydroxyproline | L-Arginine |
|  | L-Threonine |  |
|  | L-Serine |  |
|  | L-Asparagine |  |
|  | Citrulline |  |
|  | 1-Methylhistidine |  |
|  | L-Arginine |  |
|  | L-Histidine |  |
|  | Ornithine |  |

**Key:** green: the specific HILIC of the AFP plus HCC or AFP-HCC groups as compared the HGB group
