# Supplementary material for: Metabolomic Profiles for HBV Related Hepatocellular Carcinoma Including Alpha-Fetoproteins Positive and Negative Subtypes
Source: Front Oncol. 2019 Oct 15;9:1069. doi: 10.3389/fonc.2019.01069 (PMC6803550; doi:10.3389/fonc.2019.01069)
Supplement: Supplementary file 5 [file Table_5.DOCX]

**Table S5. The SCMs of HILIC column chromatography in AFP+HCC and AFP-HCC groups as compared the HGB group**

| AFP+ | AFP- |
| --- | --- |
| Propionylcarnitine | Butyrylcarnitine |
| Creatinine | Propionylcarnitine |
| L-Isoleucine | Creatinine |
| L-Carnitine | L-Isoleucine |
| Creatine | L-Pipecolic acid |
| L-Serine | L-Tryptophan |
| L-Asparagine | Betaine |
| Ornithine | 1-Methyladenosine |
|  | Creatine |
|  | 4-Hydroxyproline |
|  | L-Threonine |
|  | L-Serine |
|  | L-Asparagine |
|  | Citrulline |
|  | 1-Methylhistidine |
|  | L-Histidine |
|  | Ornithine |

**Key:** green: the common metabolites of AFP+HCC and AFP-HCC
